# Single-dose 1cp-LSD administration for canine anxiety: a pilot study

**DOI:** 10.1007/s11259-024-10542-6

**Published:** 2024-09-17

**Authors:** Luis Alberto Henríquez-Hernández, Ignacio García-Serrano, Domingo J. Quintana-Hernández, Jaime Rojas-Hernández, Elisa Hernández-Álvarez, Manuel Zumbado, Tobías Fernández-Borkel, Lucas F. Borkel

**Affiliations:** 1 Unit of Toxicology, Clinical Sciences Department, Universidad de Las Palmas de Gran Canaria, Paseo Blas Cabrera Felipe, s/n., Las Palmas de Gran Canaria, Canary Islands CP 35016 Spain; 2 Asociación Científica Psicodélica, Las Palmas de Gran Canaria, Canary Islands Spain; 3 Human Dog, Las Palmas de Gran Canaria, Canary Islands Spain; 4 Faculty of Psychology, Universidad del Atlántico Medio, Las Palmas de Gran Canaria, Canary Islands Spain; 5 Insituto-AS, Cabildo de Gran Canaria, Las Palmas de Gran Canaria, Canary Islands Spain; 6 Asociación Canaria para el Desarrollo de la Salud a través de la Atención, Las Palmas de Gran Canaria, Canary Islands Spain; 7 Faculty of Veterinary, Universidad de las Palmas de Gran Canaria, Autopista de Bañaderos a Las Palmas, 80, Arucas, Canary Islands CP 35416 Spain; 8 Institute of Psychiatry, Psychology & Neuroscience, Department of Basic and Clinical Neuroscience, Kings College, London, UK

**Keywords:** Psychedelics, LSD, 1cp-LSD, Anxiety, Dogs, Animal behavior, Animal consciousness

## Abstract

Anxiety affects 14–20% of dogs. Pharmacological treatments often fail. Psychedelics have shown to be useful for anxiety and depression in humans, but their veterinary use remains unexplored. We aimed to determine the effects of low-dose 1-cyclopropionyl-d-lysergic acid diethylamide (1cp-LSD) administered in a single dose to a dog, to observe the effect and establish the safety of the substance. The patient was a 13-year-old female dog, weighing 13 kg, mixed breed, and spayed. A total of 5 µg was administered orally, equivalent to 0.38 µg/kg. The animal has had a history of separation related behavioral problems throughout her life. To objectively assess the degree of anxiety in the dog, a validated scale was utilized. The trial was scheduled at the house where the animal lives. The owner was present throughout the experience. Informed consent was obtained prior to the assay. The trial began at 12:15 p.m. on January 10, 2024, lasting for 5 and a half hours. The response to anxiety-inducing stimuli was equally anxious during the first two hours. From that point onwards, a significant change in the animal’s behavior was observed, with no signs/mild signs of anxiety. The trial concluded without any adverse effects on the animal. The patient did not show signs of having a psychedelic experience. This is the first time that a study of this nature has been conducted and reported in the canine species. 1cp-LSD proved to be safe and exerted the desired effect on the animal’s behavior, significantly reducing the patient’s anxiety.

## Introduction

Approximately 14–20% of dogs (*Canis lupus familiaris*) are estimated to experience anxiety (Salonen et al. 2020). Anxiety inflicts substantial suffering upon the animal and its caregivers, presenting in various manifestations such as hyperactivity/impulsivity, attention deficits, aggression towards strangers, vocalization, continuous barking, and diverse compulsive behaviors, including tail-chasing, object biting, or persistent urination and defecation in inappropriate settings (Salonen et al. 2020). While a genetic component in the etiology of this behavioral disorder has been suggested (MacLean et al. 2019), there must be a significant environmental influence in the development of canine anxiety (Overall et al. 2014; Salonen et al. 2020).

To treat canine anxiety, selective serotonin reuptake inhibitors (SSRIs)– known in human medicine as antidepressants– benzodiazepines– commonly referred to as anxiolytics– and other medications (tricyclic antidepressants or Alpha2 agonists) are used (Dantas et al. 2024). Similar to human medicine, pharmacological treatments are often unsuccessful. These drugs involve chronic use, narrow safety margins, and can lead to addiction and withdrawal syndrome if abruptly discontinued (Dantas et al. 2024). Therefore, safe therapeutic alternatives are needed to enhance clinical outcomes, as the reality is that the success of pharmacological treatment, even with ethological support, is limited (Sacchettino et al. 2023). Among these new molecules are cannabinoid derivatives (CBD), but currently, their effectiveness for anxiety control in dogs is very limited (Corsato Alvarenga et al. 2023).

In the last decade, psychedelic substances have undergone a powerful resurgence (Jacobs 2021). Despite the lingering social prejudice resulting from discrediting campaigns during the period known as the ‘Acid Panic,’ these substances have demonstrated significant therapeutic potential, particularly in relation to mental illnesses, specifically depression and anxiety (Lowe et al. 2022). Moreover, psychedelics are very safe substances that do not induce addiction or leave lasting effects if consumed responsibly and at indicated doses (Schlag et al. 2022; Henriquez-Hernandez et al. 2023). Despite differences in origin, preparation, and dosage (Liechti & Holze 2022), most of these substances act similarly in the central nervous system (CNS). They all share serotonin as a common denominator, altering its homeostasis through interaction with 5-HT2A receptors (Kwan et al. 2022). However, knowledge gaps still exist to explain the effects these substances have on individuals, especially regarding the behavioral changes in their users. In the context of psychedelic therapy, recent evidence suggests that set − referred to the mental and emotional state of the individual at the time of psychedelic consumption − and setting − referred to the physical and social environment in which the psychedelic experience takes place − predict psychopathology and wellbeing (Borkel et al. 2024). Among the variables, the motivation or intention to use psychedelics emerges as a fundamental element for therapeutic success. Given that motivation is a uniquely human trait not transferable to animals, therapeutic studies involving psychedelics in animals lack will and intentionality. Nevertheless, there are documented indigenous practices in the South American continent aimed at enhancing the hunting skills of dogs through the administration of natural psychedelics found in various plants (Bennett & Alarcon 2015). This suggests that, while the level of consciousness and will in animals may not be comparable to that of the human, it may be sufficient in some species for the effects of certain psychedelic substances to be observed (Birch et al. 2020).

Lysergic acid diethylamide (LSD) has shown efficacy in treating certain behavioral disorders, such as anxiety and depression, even in microdoses (Anderson et al. 2019). Microdosing involves the administration of 10–15% of the acute effective dose, ensuring the absence of the psychedelic effects typical of these substances, as well as the absence of measurable physiological effects (Liechti & Holze 2022). Previous studies conducted in rodents have demonstrated that the administration of psychedelics in microdoses has an anxiolytic and antidepressant effect (Cameron et al. 2019). Although their beneficial effects are not replicated in all cases, such as alcohol addiction (Meinhardt et al. 2020), these substances seem to be particularly useful as anxiolytics (Horsley et al. 2018). Since the serotoninergic system in the canine species is similar to that of humans, the use of this substance could be beneficial in alleviating the anxious states experienced by dogs. The similarities between the two species extend beyond neural physiology. Dogs, like humans, develop behavioral problems such as early-development disorders, affective disorders, personality disorders, and obsessive-compulsive disorders (Goleman et al. 2019).

The aim of this study was to assess the suitability and safety of 1-cyclopropionyl-d-lysergic acid diethylamide (1cp-LSD), a legal LSD analogue, for investigating the behavioral response of a dog subjected to a single microdose of a substance known for its anxiolytic properties in humans. Given the absence of prior research in this specific context, we sought to establish a baseline for understanding canine reactions to this treatment. To our knowledge, it was the first time that this molecule was administered with therapeutic intent in this animal species, and it represents a necessary preliminary step before conducting a clinical trial. Once the effect of a single dose is known, long-term treatment can be safely initiated, allowing us to observe whether there are changes in the anxiety spectrum of dogs with this behavioral disorder. Thus, the present study reports the results and observations related to this pilot trial, and discusses the therapeutic implications of using psychedelic substances for curative purposes in animals.

## Patient and methods

### Study participant

With great altruism, a 33-year-old woman responded to the pilot program’s request to have her dog participate in the study. The patient was a 13-year-old female dog, of mixed breed, and spayed, named Lola. Despite living with the owner for a year, the animal has always been part of the family, exhibiting anxious behavior since birth. Lola’s weight on the day of the trial was 13 kg. The animal resides in a single-story house with access to a patio and outdoor gardens belonging to the house. The dog has continuous access to food and water. She coexists with another dog that does not exhibit any anxious behavior. The trial was conducted at the home where the animal and its owner reside, following the design outlined in the clinical trial. According to the dog’s medical history, she did not suffer from any pathology that could disqualify her from the trial. Informed consent was obtained from the owner before the test was performed. The animal has never received any medication to control her anxiety. The study has obtained approval from the Animal Experimentation Ethics Committee of the University of Las Palmas de Gran Canaria (Ref No. OEBA_ULPGC 02/2024). The test was supervised at all times by a veterinary ethologist and a veterinary toxicologist. The assistance of the Veterinary Clinical Hospital of the Faculty of Veterinary Medicine of the University of Las Palmas de Gran Canaria was available in case it was needed.

### Instrument for assessing anxiety

The animal has had a history of separation related behavioral problems throughout her life. To objectively assess the degree of anxiety in the dog, a validated and previously published scale was utilized (Parthasarathy & Crowell-Davis 2006). It is a questionnaire consisting of 17 questions that assess anxiety on a scale from 0 to 21 points or more, as follows: 0 to 3 points = No Separation Anxiety; 4 to 8 points = Mild Attachment Disorder (non-clinical); 9 to 15 points = Moderate Separation Anxiety; 16 to 20 points = Marked Separation Anxiety; 21 or more points = Severe Separation Anxiety. The instrument assesses anxious canine behaviors when owners are preparing to leave the house, upon return, and when the dog is alone. It also considers vocalization, barking, salivation, and compulsive behaviors such as self-mutilation or object destruction, both in terms of their presence and the intensity of these anxious behaviors. Once the dog’s basal anxiety state was established, the team of veterinarians programmed a series of stimuli that would trigger anxious responses to observe the animal’s reactions. These stimuli were selected based on the information provided by the owner about which elements provoke reactions in the dog: moving away from the animal, leaving the house, olfactory stimuli related to food, among others. These stimuli were implemented throughout the test at different time intervals, and were qualitatively categorized as follows: no anxious response, moderate anxious response, or intense anxious response.

A correlation between the psychological profiles of pet owners and the behavioral pathologies exhibited by their animals has been reported (Serpell 1996). In light of the apparent influence of owner attachment style on pet behavior (O’Farrell 1987; Rehn et al. 2017), we used an emotional attachment scale that was completed by Lola’s owner. The Non-Romantic Attachment Scale is a validated questionnaire consisting of 11 questions aimed to assess the degree of attachment not related to romantic partners of the individual, differentiating between fearful, avoidant, secure, and anxious attachment (Casullo & Fernández-Liporace 2005).

Finally, data related to the animal (age, breed, weight…), the living environment (type of house, presence of other animals…), and the owner (age, duration of cohabitation with the animal, and contact information for follow-up) were collected. The questionnaire included two additional questions: (i) Are you clinically diagnosed with anxiety/depression? and, (ii) Do you take anxiolytics or any medication for anxiety/depression?

### Characteristics and dosage of 1cp-LSD

1cP-LSD (1-cyclopropionyl-d-lysergic acid diethylamide) is a semi-synthetic psychedelic substance belonging to the class of lysergamides. It is an analogue of LSD and has been available for research since July 2019 (Brandt et al. 2020). Similar to other lysergamides, it is likely that 1cP-LSD interacts with a wide range of monoamine receptors, such as adrenergic and dopamine receptors, in addition to acting as an agonist of serotonin 5-HT receptors (Nichols 2016). The substance hydrolyzes to LSD through the action of blood carboxylesterases, giving rise to the compound. Therefore, it behaves as a prodrug (Brandt et al. 2020). However, nowadays it is unclear whether the nature of the 1-acyl substituent leads to differences in pharmacokinetic parameters compared to other semi-synthetic psychedelic substances, including the formation of LSD in vivo.

**Fig. 1:**
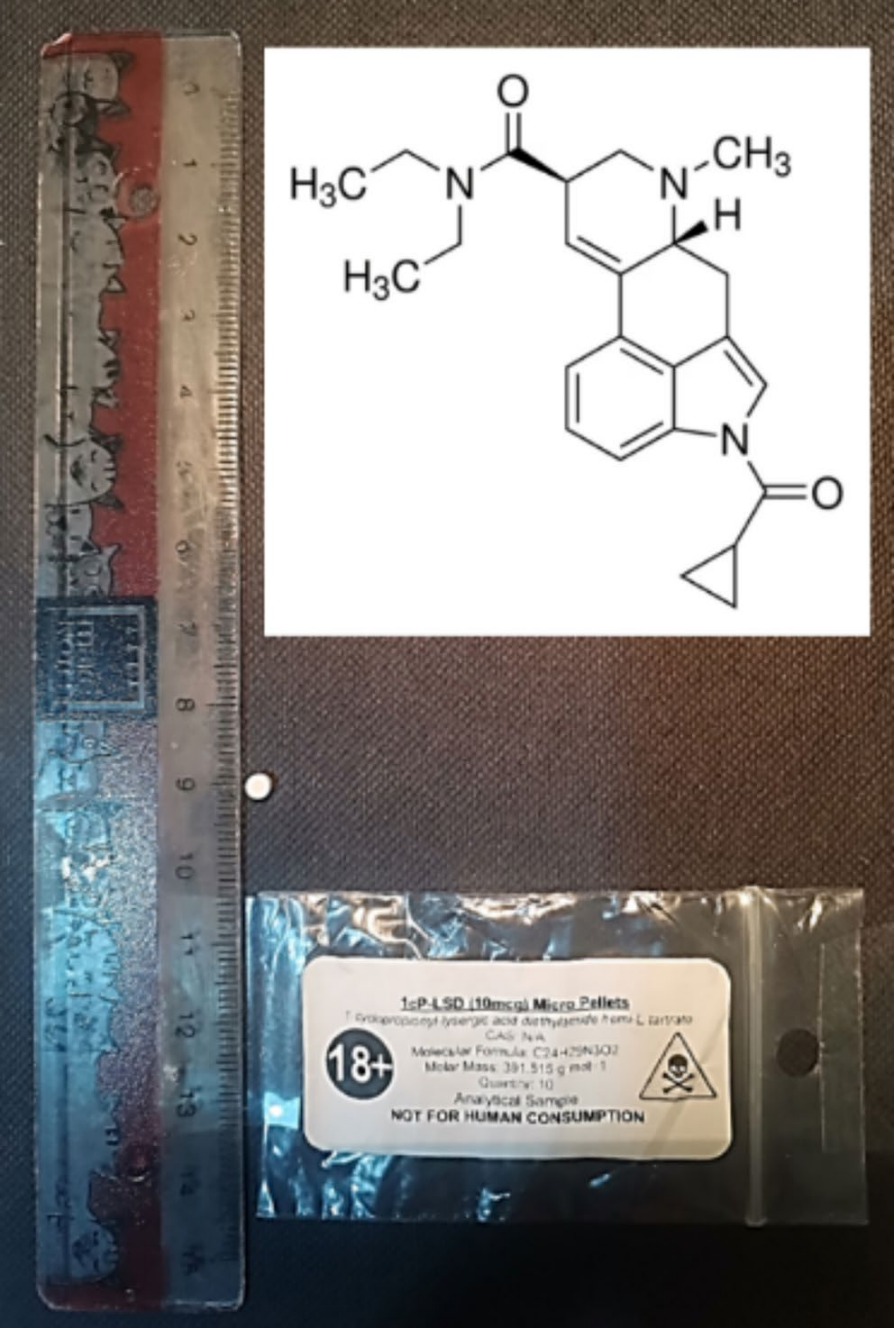
Image showing a 10 µg pellet of 1cp-LSD and its original packaging. The chemical structure of the molecule is displayed in the top right corner

1cp-LSD was legally acquired through an online supplier. Each pellet contains 10 µg of the active compound and is designed to be cut and dosed at 5 µg (Fig. 1). Therefore, a total of 5 µg was administered orally to the subject, equivalent to 0.38 µg/kg. Translated to humans, this represents a dose of 30.7 µg (for an 80-kilogram person), slightly exceeding the upper limit of a typical microdosing range (5–25 µg) (Liechti & Holze 2022).

### Procedure

The administration of the substance took place on January 10, 2024, at 12:15 PM. 5 µg of 1cP-LSD (half a pellet) was administered, disguised in a piece of ham. The dosage was prepared by the veterinary toxicologist, following the manufacturer’s recommendations for its handling. According to the owner’s report, the dog had consumed a meal approximately 90 min prior to the initiation of treatment. The trial was scheduled at the house where the animal and its owner live: a countryside residence in a rural setting with access to a patio and outdoor gardens belonging to the house. In addition to the owner, there was another family member as well as two members of the research team: a toxicologist and an ethologist, both veterinarians. Given the unfamiliarity of the observers to the animal, a one-hour acclimation period was implemented before drug administration. The dog demonstrated friendly behavior throughout the interaction. The role of the research team was purely observational, with no intervention on the animal beyond a physical examination to evaluate the effect of the substance, primarily mydriasis. The two investigators continuously observed the animal’s behavior, working closely with the owner to identify any abnormal behaviors. Both the owner and the family member were present throughout the trial. While stress triggers were indeed explored during the trial (scheduled absences, specific sounds), everything was done with the prior approval of the animal’s owner. The anxiety and emotional attachment questionnaires were administered on that day. After the trial was concluded, communication was maintained with the owner for the remainder of that day and during the following seven days.

## Results

The entire experience was documented in a video accessible through this document (available at: https://www.youtube.com/watch?v=Eb46Jcp6HM8). The subject’s score on the anxiety scale was 29 points, indicating severe anxiety. Anxious behaviors, including whining, barking, crying, and furniture destruction, were notable when the owners left the house and while they were away. An anxious behavior was reported in terms of effusive greeting when the owners return home, and compulsive following wherever they go within the home context. Extensive biting and scratching on home furniture were also reported when the animal was left alone. There were no reported compulsive urinations or defecations, nor excessive salivation.

The analysis of the owner’s questionnaire reveals a fearful/avoidant attachment profile with a high anxious component, reporting the following scores: 9 out of 12, 8 out of 12, and 7 out of 12 possible points for fearful, anxious, and avoidant attachment subscales, respectively. The score for secure attachment was the lowest, obtaining 4 out of 8 points. A fearful-avoidant attachment profile with an anxious component suggests an insecure connection, marked by fear of intimacy and simultaneous need for closeness.

**Fig. 2:**
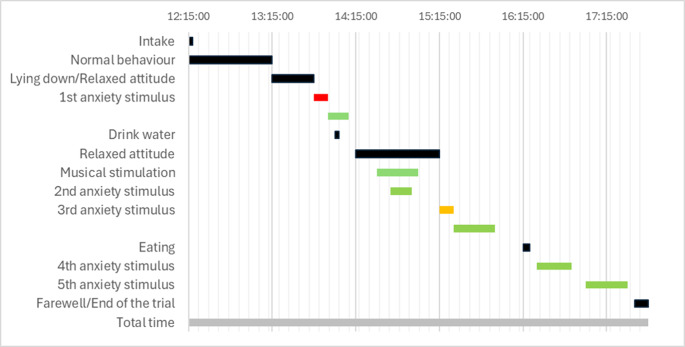
Gantt chart depicting the temporal progression of the experience. Green represents no anxious response, orange represents moderate anxious response, and red represents intense anxious responses to administered stimuli

Thirty minutes post-administration of the substance, Lola’s behavior was completely normal, according to her owner’s report (Fig. 2). In this sense, the owner’s observations were crucial to complement the observations recorded by the specialized personnel who supervised the test. She interacted with her surroundings, wagging her tail continuously, and exhibited a docile and engaged demeanor. After an hour, the animal lay down to sunbathe, not falling asleep, with the owner not observing anything abnormal. Minutes later, she sought shelter inside the house. Upon observation, she got up without issues and interacted normally. Ninety minutes after drug administration, we subjected Lola to an anxiety stimulus. The owner and the family member left the house, and the dog exhibited typical anxious behavior of barking and whining. Based on the owner’s report, the observed behavior was consistent with the typical anxiety’s dog response. It should be emphasized that the anxious behavior is particularly evident in the absence of the primary caregiver, notwithstanding the presence of other individuals (the observers, in this particular instance). After a few minutes, Lola greeted them warmly, although they reported it might have been ‘less than usual.’ Minutes later, the owners went to another part of the house. They expected the animal to follow them, as it consistently exhibited that anxious behavior, something that was recorded in the anxiety questionnaire. However, the animal did not display such behavior. She did not even draw attention from the owners through barking or whining. Lola drank water for the first time at that point. The owners expressed surprise at this behavior.

After two hours, exhibiting a temporal pattern of the effect similar to that observed in humans exposed to this substance, Lola appeared calm (Fig. 2). She climbed into the chair by herself. At that moment, specific music for the psychedelic experience was played. The animal appeared relaxed and showed no signs of discomfort or aversion to the sound. After a few minutes, we repeated the anxious stimulus: the owner left the house. The patient did not follow her, did not bark or vocalize. Minutes later, she came back. The dog recognized her, wagging her tail, and greeted her calmly without leaving the couch. Lola’s behavior had changed, something very evident to her owner, who expressed it. The researchers present at the trial did not observe mydriasis, which is one of the most evident signs of an LSD toxic effect.

Three hours after the administration, the anxious stimulus was repeated (Fig. 2). After leaving the house, the dog left the couch and barked minimally at the door. We returned her to her spot, and she stayed there for a few minutes until the owner returned. At that moment, the animal did not show signs associated with its anxious behavior, such as whining, barking, or effusive greetings. The animal ate after 4 h from the start of the experience. She was observed going up and down stairs, exploring, wagging her tail, and interacting with the environment. No anxious reaction was observed to stimuli such as the owners being in the kitchen preparing food. Abdominal movement associated with breathing was detected, corresponding to a whimper that did not fully manifest. No dyspnea or any adverse effects were observed. The animal did not sleep at any point.

Lola remained calm while her owners ate. This occurred 5 h after administration. No anxious behaviors related to the owners’ mealtime, which frequently happened, were observed. After 5.5 h, the pilot trial was concluded (Fig. 2) without any adverse effects on the animal: growls or barks indicative of an altered emotional state, aggressive behaviors, loss of consciousness, or others. Despite the slightly higher dose than that typically used for microdosing, the animal did not show signs of having had psychedelic experience (stumbling, howling, restlessness, fear), not even at a physical level (mydriasis). Consistent communication was maintained with the animal’s owner for the following 24 h, ensuring that cognitive recovery was ongoing and no veterinary intervention was required. No clinically significant events were reported. To assess the treatment’s effect on anxiety modulation, it’s essential to establish a continuous microdosing protocol over time (1–3 months), akin to human trials, which will be explored in a clinical trial.

In summary, this pilot study has shown that the tested dose of 1cp-LSD is safe for dogs: no adverse effects were observed (agitation, mydriasis, loss of consciousness, vomiting, confusion, or panic attacks) and it produced the expected effect compared to what occurs in human microdosing (tranquility, relaxation, interaction with the environment, and a similar temporal development around 5–6 h).

## Discussion

In this study, 5 µg of 1cp-LSD was administered to a 13-year-old, 13-kilogram mixed-breed dog named Lola, with a history of severe anxiety, according to the canine anxiety assessment scales used. As reported above, specific symptoms of anxiety related to separation from her owner were mitigated, such as reduced barking, crying, and overall distress. Also, symptoms of separation anxiety and anxiety caused by exposure to specific stressors like the sound of doorbells and machinery were not present during the experience. Nevertheless, the continuous presence of two observers during the treatment could have influenced the animal’s behaviour. To mitigate this potential bias, the owner’s report of the animal’s behaviour was crucial for drawing objective conclusions from the observations. The present study suggests that further studies with 1cP-LSD for anxiety reduction in dogs are warranted. Given the established relationship between human and animal behaviour in the context of pets, we also believe that further studies should not only clearly identify animal-individual specific stressors (e.g. sounds), changes in behaviour before, during and after treatment, qualitative observations (e.g. body language, interaction with owner and other animals) and drug tolerance; but also the potential effect of attachment to owners (Parthasarathy & Crowell-Davis 2006) and owner to pet stress transmission (Harvie et al. 2021). In the present study, dog’s owner exhibited a fearful-avoidant profile with an anxious component, suggesting an insecure connection characterized by a fear of intimacy and a simultaneous need for closeness. The potential influence of this on the animal’s behaviour needs further investigation. It has been reported that owners may attribute improvements in their own symptomatology to their pet’s condition. Conversely, transient elevations in owner anxiety can significantly impact canine anxiety levels (O’Farrell 1987; Pereira et al. 2021). Therefore, future studies should consider the presence of behavioural disturbances in owners of dogs with separation related behavioral problems.

As shown in the results, the anxiolytic effects of the substance were temporary as baseline behaviour and symptoms returned 4–5 h after the intake. This observation is crucial for understanding the duration of 1cP-LSD’s effectiveness in this context. For this reason, further studies should implement a specific microdosing protocol to assess its (potential) cumulative therapeutic.

effect, as has been done in human studies (Murphy et al. 2023).

The observations of patient 0 being less impulsive, more relaxed, and less prone to fear or anxiety after administration of 1cP-LSD suggest an alteration in emotion processing and an attentional shift away from stressors. This result is consistent with previous animal trials with LSD that report symptoms of sedation, depression, excitation, changes in behaviour and hallucinations (Oster et al. 2023). Other alterations have been reported in dogs that are regularly administered with several psychedelics in order to improve their hunting abilities in the context of indigenous practices (Bennett & Alarcon 2015). Serotonin agonists like ergot-like alkaloids, N,N-Dimethyltryptamine, psilocybin/psilocin and 5-meth-N, N-dimethyltryptamine which carry hallucinogenic effects in both human and animal models (McKenna et al. 1984; Wink 2000), are supposedly associated to the increase of hunting effectiveness in both humans and animals. This is consistent with the evidence of psilocybin triggering thalamic down-regulation and frontal hypermetabolism, which has been suggested to induce drug-dependent synaesthesia (Stevenson & Tomiczek 2007). This neurological condition is defined as the coupling of two or more bodily connected senses, induced by the reduction of sensory feedback pathways, mechanism which might be a causal factor for altered consciousness, manifested in the form of enhanced olfactory, auditory and visual perception. Due to the shared characteristics of these alkaloids with 1cP-LSD, alterations in perception such as synaesthesia and sensory enhancement should not be discarded for patient 0. Therefore, we consider that further research into the psychoactive effects of 1cP-LSD microdoses should be further explored in a clinical trial with a larger sample. Whereas the exact mechanism by which 1cP-LSD reduces anxiety is unknown, parallels from human studies with similar psychedelic substances could be drawn. In humans, LSD, psilocybin and other classic psychedelics have been demonstrated to act on serotonin 2 A receptors (5-HT_2A_), which play a significant role in mood and anxiety regulation (Nichols 2016). Further clinical trials could show how similar mechanisms might be at play in dogs and other mammals, and draw parallels between human and non-human animals regarding attentional and emotional regulation, memory access and memory reconsolidation, among other things.

Additionally, our case study also suggests that research on animal altered experiences could shed light on fundamental aspects of animal consciousness. Understanding how such substances influence the canine cognition and behaviour could offer insights into the broader field of animal experience, bridging the gap between neurophysiological changes, observable behavioural outcomes, and the difficult topic of animal consciousness. Previous studies have discussed the emergence of consciousness in the evolutionary timeline proposing the use of general anaesthesia to study the resulting emergence process of consciousness as a model to assess the development across species (Mashour & Alkire 2013). These approaches delve into the role of cortical and subcortical regions involved in the phenomenon of consciousness in non-human species, which tie in with our case by providing a broader context on how consciousness and sensory perception are interconnected across species. A model based on psychedelics could be a different approach to explore neural correlates which underlie consciousness emergence in humans and non-human animals as well as fundamental phenomenological aspects of conscious experience shared by different species.

## Conclusions

According to our knowledge, this is the first time that a pilot trial of this nature has been conducted and reported in the canine species. The 1cp-LSD proved to be safe under these conditions (appropriate setting) and exerted the desired effect on the animal’s behaviour, significantly reducing the patient’s anxiety. The duration of the experience was comparable to that reported in humans, with the peak effect observed at 2–3 h and a gradual return to baseline from 5 h onwards. These findings will allow for the safe conduct of future studies investigating the potential utility of 1cp-LSD microdosing for the treatment of dogs with separation related behavioral problems.

## Data Availability

No datasets were generated or analysed during the current study.
